# Evaluating the Efficacy of Prucalopride, a 5-HT4 Agonist, in Managing Antipsychotic-Induced Constipation: A Prospective Randomized Controlled Trial Conducted at Chronic Psychiatric Rehabilitation Facilities on Corfu Island

**DOI:** 10.1192/j.eurpsy.2024.1447

**Published:** 2024-08-27

**Authors:** P. Argitis, M. Peyioti, O. Pikou, M. Demetriou, A. Karampas, S. Karavia, Z. Chaviaras

**Affiliations:** ^1^Psychiatric; ^2^Dermatology, General Hospital of Corfu, Corfu, Greece

## Abstract

**Introduction:**

Achieving successful stabilization in patients with mental disorders often requires the administration of multiple antipsychotic medications, with the increasing prevalence of clozapine in cases resistant to other treatments. Constipation emerges as a particularly troublesome side effect, gradually progressing into a chronic state of gastrointestinal dysfunction, often accompanied by recurrent episodes of paralytic ileus of varying severity. Prucalopride, a 5-HT4 agonist, selectively targets receptors within the intestinal system.This interaction induces muscular contractions and promotes chloride secretion. Literature suggest its potential efficacy in managing constipation induced by clozapine. In light of these observations, we designed and will conduct a randomized controlled trial to evaluate the effectiveness of prucalopride in alleviating constipation in patients who had shown limited responsiveness to conventional laxatives or other conservative treatments

**Objectives:**

The primary objective of this article is to present the methodology of a randomized control trial assessing the efficacy of prucalopride in the treatment of constipation among patients with mental disorders

**Methods:**

The study will enroll 60 adult patients with mental disorders who will require more than two antipsychotic medications, including clozapine, for stabilization, and who will be experiencing constipation as a side effect

To ensure the validity of the study, the following additional inclusion criteria will be applied:

- Patients will have no severe acute medical conditions
- Patients will have no history of malignancy
- Patients will have no severe respiratory or cardiac diseases
- Patients will have negative results from an endoscopic evaluation of the large bowel, ruling out conditions such as irritable bowel syndrome, ischemic colitis, inflammatory bowel disease, or malignant neoplastic disease

Following the screening process, the patients will be randomly assigned to one of two treatment groups:

**Prucalopride Group:** Patients in this group will receive prucalopride for the treatment of refractory constipation

**Conservative Treatment Group:** Patients in this group will continue with conservative treatments.The treatment’s success will be determined based on specific endpoints:

- Normalization of bowel movements, characterized by having more than five bowel movements per week
- Resolution of symptoms related to gastrointestinal dysfunction, including pain, bloating, defecation difficulties, and paralytic ileus

**Results:**

Following the conclusion of the study, data from both groups will be meticulously collected and subjected to rigorous statistical analysis to identify differences in treatment outcomes between these two therapeutic approachs

**Conclusions:**

The detailed findings will be presented in a forthcoming article

**Disclosure of Interest:**

None Declared

